# The Effect of Lithium on the Budding Yeast *Saccharomyces cerevisiae* upon Stress Adaptation

**DOI:** 10.3390/microorganisms10030590

**Published:** 2022-03-09

**Authors:** Patrick Reith, Svenja Braam, Niek Welkenhuysen, Sarah Lecinski, Jack Shepherd, Chris MacDonald, Mark C. Leake, Stefan Hohmann, Sviatlana Shashkova, Marija Cvijovic

**Affiliations:** 1 Department of Mathematical Sciences, University of Gothenburg, 412 96 Gothenburg, Sweden; reith@chalmers.se (P.R.); braam@chalmers.se (S.B.); niek@chalmers.se (N.W.); 2 Department of Mathematical Sciences, Chalmers University of Technology, 412 96 Gothenburg, Sweden; 3 Department of Biology and Biological Engineering, Chalmers University of Technology, 412 96 Gothenburg, Sweden; hohmann@chalmers.se; 4 Department of Physics, University of York, York YO10 5DD, UK; sarah.lecinski@york.ac.uk (S.L.); jack.shepherd@york.ac.uk (J.S.); mark.leake@york.ac.uk (M.C.L.); 5 Department of Biology, University of York, York YO10 5DD, UK; chris.macdonald@york.ac.uk

**Keywords:** lithium, osmotic stress, macromolecular crowding, protein aggregation, long-term survival, yeast

## Abstract

Lithium salts are used in the treatment of mood disorders, cancer, and Alzheimer’s disease. It has been shown to prolong life span in several phyla; however, not yet in budding yeast. In our study, we investigate the influence of lithium on yeast cells’ viability by characterizing protein aggregate formation, cell volume, and molecular crowding in the context of stress adaptation. While our data suggest a concentration-dependent growth inhibition caused by LiCl, we show an extended long-term survival rate as an effect of lithium addition upon glucose deprivation. We show that caloric restriction mitigates the negative impact of LiCl on cellular survival. Therefore, we suggest that lithium could affect glucose metabolism upon caloric restriction, which could explain the extended long-term survival observed in our study. We find furthermore that lithium chloride did not affect an immediate salt-induced Hsp104-dependent aggregate formation but cellular adaptation to H_2_O_2_ and acute glucose starvation. We presume that different salt types and concentrations interfere with effective Hsp104 recruitment or its ATP-dependent disaggregase activity as a response to salt stress. This work provides novel details of Li^+^ effect on live eukaryotic cells which may also be applicable in further research on the treatment of cancer, Alzheimer’s, or other age-related diseases in humans.

## 1. Introduction

Lithium is listed on the WHO model list of essential medicines as a drug used for the treatment of mood disorders, such as bipolar disorder and depression [1,2,3,4]. It has been suggested that LiCl could induce autophagy [5], which is a key cellular process in the inhibition of cancer [6]. Recently, it has also been shown that lithium inhibits proliferation of prostate and colorectal cancer cell lines [7].

Salt ions cause osmotic and ionic stress. Chemically, Li^+^ ions are similar to Na^+^; however, they have been reported to have a cytotoxic effect at much lower concentrations compared to sodium [8]. Lithium ions have been shown to inhibit metabolic enzymes, such as phosphoglucomutase, which catalyse the reaction of glucose-1-phosphate to glucose-6-phosphate [9], and inositol monophosphatase involved in the formation of *myo*-inositol, the precursor for all inositol compounds from glucose-6-phosphate [10]. These findings indicate that Li^+^ impacts glucose metabolism.

During the last decade, the possibilities for a lithium-based treatment of Alzheimer’s disease and other neurodegenerative disorders have been investigated [11,12]. Alzheimer’s disease is associated with misfolding and aggregation of the Tau protein [13]. Most studies on lithium in the context of neurodegenerative disorders presume LiCl inhibits glycogen synthase kinase-3, leading to a reduction in Tau protein hyperphosphorylation and aggregation [14]. The formation of protein aggregates is one of the hallmarks of many neurodegenerative diseases [15]; however, the general effect of LiCl on protein aggregation is still unclear.

Furthermore, lithium has been associated with lifespan-extending effects in different model organisms, such as the nematode *Caenorhabditis elegans*, the fly *Drosophila melanogaster* fly, and the fission yeast *Schizosaccharomyces pombe* [16]. However, the exact role of lithium ions in the long-term survival of these organisms remains to be elucidated.

In this work, we explore the effects of Li^+^ ions on the budding yeast *Saccharomyces cerevisiae* adaptation to stress in the context of cellular viability, volume, molecular crowding, and aggregate clearance. We compare Li^+^ to the chemically similar and abundant Na^+^ ion, which is well characterised in its role in salt homeostasis. Our results suggest that the toxic effect of LiCl is dependent on glucose availability. We also show that LiCl in combination with caloric restriction might lead to an increase in long-term viability.

## 2. Materials and Methods

### 2.1. Yeast Strains and Media

Yeast strains were cultivated at 30 °C, 200 rpm in Synthetic Defined Media (SDM: 1x Yeast nitrogen base, 1x amino acid mix, Formedium Ltd., UK; 5 g/L ammonium sulphate) supplemented with 2% (*w/v*) glucose (Merck), unless specified otherwise. For the viability assay, standard YPD agar plates were used (10 g/L yeast extract, 20 g/L peptone from meat, 2% (*w/v*) glucose, 20 g/L agar). Stress conditions were established by adding NaCl (Merck, EMSURE), LiCl (Sigma Aldrich) or H_2_O_2_ (Sigma Aldrich) to the appropriate concentration.
Strains used in this study.**Name of the Strain****Genotype****Source****BY4741***MATa his3Δ1 leu2Δ0 met15Δ0 ura3∆*Hohmann collection**Hsp104-GFP***HSP104*-*GFP::HIS3* in BY4741Nyström collection**crGE**pRS303*_TEF1pr_CrGE::HIS3* in BY4741Boersma Lab [17]

### 2.2. Growth of Yeast Cells under Salt Stress Conditions

The BY4741 strain was taken from YPD agar plate culture and grown overnight in 5 mL YPD. Precultures were harvested by centrifugation and washed in 5 mL of SDM and OD₆₀₀ measurement was performed. Next, cells were inoculated to the appropriate testing conditions to an OD₆₀₀ ≈ 0.05 into BioScreen 100-well microplates. The growth assay was performed with the BioScreen Analyser C (Thermic Labsystems). Throughout the experiment, an incubation temperature of 30 °C was used. The read-out plates were subjected to continuous shaking, which was seized 30 sec before each reading. OD₆₀₀ measurements were taken every 30 min for 72 h. Testing conditions were run in triplicates.

### 2.3. Western Blotting

Activation of the high-osmolarity glycerol (HOG) pathway was assessed by the phosphorylation status of Hog1, as described previously [18]. A single colony of strain BY4741 was inoculated into 15 mL of SDM, cultured overnight, and used to inoculate 50 mL of fresh medium to OD₆₀₀ ≈ 0.2. When the culture reached OD₆₀₀ ≈ 0.5–0.7, it was split into 4 flasks (12 mL each). Different stress conditions were applied to each flask by adding 3 mL of an appropriate medium prewarmed to 30 °C with or without salt to obtain the final concentrations of: no salt; 1 M NaCl, 250 mM NaCl, 200 mM LiCl. Samples were taken directly before, immediately after, and 5, 15, 30, 45, 60, 75, 90 min after addition of the medium.

For the sampling, 1 mL of the culture was mixed directly with 190 μL of fixation buffer (80% Tri-chloro-acetic acid, 100 μg/mL Cycloheximide). The cells were then pelleted at 13k x g, 10 sec and washed twice with 1 mL of wash buffer (600 mM Tris-HCl, 40% acetone). The proteins were extracted with 60 μL of extraction buffer (90 mM Tris-HCl, pH 6.8; 200mM DTT; 3% SDS; 13% glycerol; 2% β-mercaptoethanol; 1x PhosSTOP™, Roche; 1x cOmplete™, EDTA-free protease inhibitor cocktail, Roche; 0.005% Bromophenol Blue), 20 μL of glass beads in a Thermomixer (Eppendorf) at 98 °C, 1400 rpm, 10 min. The samples were centrifuged at 13k g for 10 min. The protein extract from the supernatant was then resolved via SDS-PAGE on 4–20% Mini-PROTEAN^®^ TGX™ Precast Protein Gels, 200 V for 35 min, and transferred onto a low fluorescence PVDF membrane (Trans-Blot Turbo Transfer System, Bio-Rad). The proteins were detected as previously described [18] using primary Anti-Hog1 Antibody (D-3), Santa Cruz Biotechnology; Phospho-p38 MAPK (Thr180/Tyr182) Antibody, Cell Signaling Technology; Phospho-p44/42 MAPK (Erk1/2) (Thr202/Tyr204) Antibody, Cell Signaling Technology and secondary IRDye^®^ 800CW Goat anti-Mouse Antibody (LI-COR Biosciences); IRDye^®^ 680RD Goat anti-Rabbit Antibody (LI-COR Biosciences). Blots were imaged using a ChemiDoc MP (Bio-Rad). The signals of total Hog1 and phosphorylated Hog1 (Hog1-P) were measured in ImageJ/Fiji using the Gels tool. Lanes were selected and an *X*-axis projected intensity plot was generated. The signal peaks referring to Hog1 total and Hog1-P were isolated from the background and the integrated intensity was measured as signal intensity values. The signals were normalised to the pre-salt timepoint and the ratio of Hog1-P to Hog1 total was calculated.

### 2.4. Cell Volume Recovery Assessment

To assess the cell volume recovery upon osmotic shock, BY4741 cells were grown in SDM overnight, diluted to OD₆₀₀ ≈ 0.2 and seeded onto a glass bottom dish (WillCo Wells). To obtain a desired condition (no salt; 1 M NaCl, 250 mM NaCl or 200 mM LiCl), an appropriate medium with or without salt was added to the dish. The cells were imaged right before the addition of the conditioning medium, right after and then every 15 min for a 90 min timespan. The resulting images were used to measure the cell circumference in ImageJ/FIJI as an indicator of cell volume.

### 2.5. Aggregate Formation upon Salt Stress

BY4741 Hsp104-GFP cells were cultured overnight in SDM. The cells were then seeded onto a poly-L-lysine coated glass bottom dish and the conditions were reached as indicated above. The cells were imaged right before and immediately after addition of the conditioning media and at the following timepoints: 5, 15, 30, 45, 60, 90 min. Stacks of ten images were taken over a total axial distance of 7 µm on three fields of view per condition on a Leica DMi8 inverted fluorescence microscope (Leica microsystems, Wetzlar, Germany) equipped with a HCX PL APO 40 ×/1.30 oil objective (Leica Microsystems), Lumencor SOLA SE (Lumencor, Beaverton, OR, USA) led light and Leica DFC9000 GT sCMOS camera (Leica Microsystems) with 450/490 nm excitation, dichroic 495 nm, and 500/550 nm emission.

The number of fluorescent foci per cell was determined in ImageJ/FIJI.

### 2.6. Crowding Measurements

BY4741 S. cerevisiae yeast cells expressing the CrGE crowding sensor [17,19] genetically integrated at the HIS locus [19,20] were grown in SDM lacking histidine to an OD₆₀₀ of 0.4 (mid-log phase) and centrifuged at standard speed (3000g) before re-dilution by a factor of 2 to a final OD₆₀₀ of 0.2 for imaging.

Glass-bottom dishes (35 mm Ibidi GmbH) were coated with 1 mg/mL stock concentration of Concanavalin A (ConA) by incubation with 300 µL of stock solution for 5 min before three washes with 2 mL ultrapure water. Cells were left for 5 min to adhere at room temperature on the ConA treated glass-bottom dishes before washing three times with 2 mL SDM-His to remove unattached cells. Osmotic shock was achieved on the microscope via media exchanges using microfluidics as described previously [21]. Confocal microscopy was performed on a commercial LSM880 Zeiss microscope equipped with an Airyscan module, Nikon 63x Plan-Apochromat oil immersion objective lens NA 1.4, and a pinhole size 4.61 AU (Airy-Units) to give a 3 μm × 3 μm confocal section. mCerulean was excited by a 458 nm laser and fluorescence imaged through a 463–500 nm bandpass filter. For FRET measurements, the sample was excited by the 458 nm laser but using a 525-606 nm bandpass emission filter. The laser was set at 1.5% of maximum power for all imaging. Cells were imaged at room temperature 10 min and then 5 min prior to media exchange, then every 5 min for 90 min after exposure to stress media (SDM supplemented with 1 M NaCl, 250 mM NaCl, or 200 mM LiCl). All media were pre-tempered at 30 °C. Image analysis was performed using YeastSpotter a deep learning segmentation tool [22] integrated in an analysis pipeline coded in Python3, which extracted mean intensities per pixel in the FRET and donor channels for each detected cell, and mapped calculated values for ratiometric FRET across the cellular volume.

### 2.7. Microscopy of Aggregates Formed in Cells Exposed to Different Stressors after Pre-adaptation to Salt

Yeast cells of the BY4741 Hsp104-GFP strain were inoculated into 50 mL of SDM and grown overnight, then diluted to an OD₆₀₀ of 0.8 in 100 mL SDM and incubated for 30 min, 200 rpm, 30 °C. After this incubation, four samples of 20 mL were prepared and diluted to an OD₆₀₀ of 0.4 in standard SDM as a control and SDM supplemented with the respective salt to reach a final concentration of 200 mM LiCl, 250 mM NaCl, or 1 M NaCl for preadaptation, and incubated for 2 h at 30 °C, 200 rpm before applying a secondary stress.

Before the application of heat stress, hydrogen peroxide, or glucose downshift, a sample of 1.8 mL was taken from each culture.

For the application of hydrogen peroxide, a 10 mM solution of H_2_O_2_ (Sigma-Aldrich, St. Louis, MO, USA) was freshly prepared and added to each cell culture to a final concentration of 1 mM. The cells were incubated at 30 °C, 200 rpm, and samples of 1.8 mL were taken every 15 min up until 90 min after adding of H_2_O_2_.

For heat shock, 10 mL of cultures were transferred to a 38 °C water bath in 15 mL screw cap tubes and samples were taken every 15 min until 90 min after transfer to the water bath.

For glucose downshift, cells incubated in salt medium were centrifuged (1500× *g*, 5 min) and resuspended in the same amount of medium with the respective salt condition but with 0.05% (*w*/*v*) glucose. Immediately after the exchange of medium, samples were taken from the culture. Further, samples were taken every 15 min until 90 min after the shift to low glucose.

Each sample was fixed by adding a final concentration of 3.7% (*v*/*v*) formaldehyde, incubated for 30 min at ambient temperature, washed twice in PBS (6000× g, 20 s), and resuspended in 20 µL of sterile PBS (pH 7.4). Samples were stored at 4 °C for up to 24 h before imaging.

Stacks of five images were taken over a total axial distance of 3 µm on three fields of view per condition on a Leica DMi8 inverted fluorescence microscope (Leica Microsystems) equipped with a HCX PL APO 40×/1.30 oil objective (Leica Microsystems), Lumencor SOLA SE (Lumencor) led light and Leica DFC9000 GT sCMOS camera (Leica Microsystems) with 450/490 nm excitation, dichroic 495 nm and 500/550 nm emission, exposure time 200 ms. Images were analysed using the Cell counter plugin in the FIJI distribution of ImageJ after performing a maximum intensity Z-projection.

Per time point and condition, 100 cells +/− 10 per field of view were counted. The numbers of cells with 0, 1, 2, or > 2 aggregates were counted. The values obtained were averaged and expressed as fraction of cells with aggregates of the total averaged number of cells counted.

### 2.8. Cell Density Correlation and Survival Assay

A single BY4741 colony was inoculated into 3 mL of SDM and incubated at 30 °C, 200 rpm, overnight, then transferred into 50 mL of SDM and incubated for 1 day. From this preculture, cells were transferred into SDM with different concentrations of salt and glucose to test long-term cell survival. The amount of cells that is needed for a volume of 10 mL of the respective salt medium with a starting OD₆₀₀ of 0.1 was harvested by spinning down the corresponding volumes of the preculture (1500× g, 5 min) and resuspending these cells in 10 mL each of the respective test media with the following salt and glucose concentrations: SDM with 250 mM NaCl, 1 M NaCl, 50 mM LiCl, 200 mM LiCl, SDM with 0.5% (*w/v*) glucose with and without 200 mM LiCl, as well as 10 mL of SDM without any salt as a control. Cells were incubated in 50 mL screw-cap falcon tubes, 30 °C, 200 rpm for 1 day, and a 500 µL sample for each condition was taken (day 1). Samples were taken on day 4, 7, 10, 13, 16, and 19.

For each sample, the OD₆₀₀ was measured, adjusted to 0.2 and series of 10-fold dilutions were prepared to a final density of 0.2 × 10^−5^. Three dilution steps, 100 µL each, were spread onto YPD plates, incubated for 2 days at 30 °C and scanned for further analysis. For day 1, the three highest dilutions were used. For the days after, selection of dilutions was adjusted based on growth observed from the previous timepoint. Three biological replicates were made per condition and timepoint, two technical replicates were performed.

Analysis of colony forming units (CFU) was done using the Fiji distribution of ImageJ with an in-house macro that automates the use of the plugin colony counter.

Since we tested one strain in different media for this assay, we accounted for effects on cells that influence OD₆₀₀ measurements and therefore could lead to an over- or underestimation of the CFU count. We employed a cell density correction factor (CDCF) that was calculated as follows:

For day 1, CFU counted per plate were multiplied to fit CFU/per 1 OD₆₀₀. The values obtained for dilution factors were averaged and normalised by dividing the respective condition at day 1 by the control condition at the same day. This was calculated per biological and technical replicate, so that for each biological/technical replicate, the control condition at day 1 has a correction factor value of 1, whereas for a higher number of viable cells per 1 OD₆₀₀ unit, the value would be higher. The value obtained was used to divide with the values obtained for CFU/(1 OD₆₀₀) unit for day 4 to 19.

## 3. Results

### 3.1. Salt Stress Reduces the Growth Rate and Maximal Cell Density

**Figure 1:**
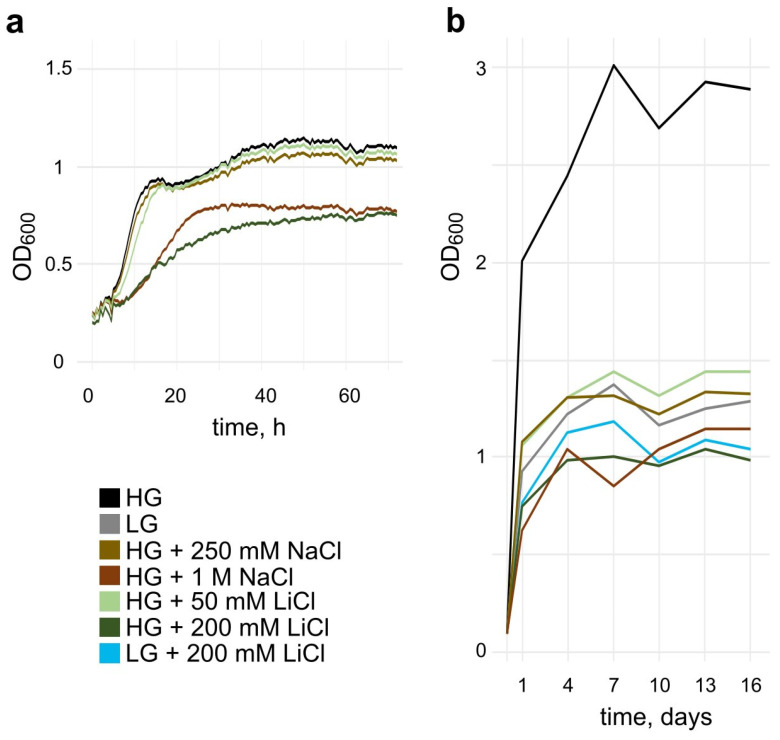
Growth curves of *S. cerevisiae* strain BY4741 in SDM, supplemented with 2% glucose (unless indicated otherwise). Growth conditions were applied at timepoint 0. HG = High Glucose, 2% (*w/v*); LG = Low Glucose, 0.5% (*w/v*). Experiments were run in triplicates and plotted as an average. (**a**): Optical density measured from cultures grown in a honeycomb plate with a Bioscreen C instrument for 71 h. (**b**): Optical density measurement with a spectrophotometer of samples taken from cultures grown in 50 mL Falcon tubes over 16 days.

To investigate the effect of LiCl and NaCl on the yeast cells, we first measured the growth profile of cultures cultivated with different salt concentrations using a Bioscreen C instrument (Figure 1a). The presence of 250 mM NaCl or 50 mM LiCl did not affect cellular growth. Lower concentrations of the salts did not show a growth inhibition either (data not shown). 1 M NaCl or 200 mM LiCl resulted in a reduced growth rate and a lowered maximal OD₆₀₀ of ca. 60% in comparison to the standard condition. Interestingly, while 250 mM NaCl did not affect growth, the 200 mM LiCl condition with similar osmolality caused this decline in growth.

In addition, we looked at the growth of yeast cultures observed during a period of 16 days in culturing tubes during a survival assay with different glucose concentrations with or without the addition of NaCl or LiCl (Figure 1b). Compared to control conditions, we found all conditions grew slower and plateaued at a reduced (30–50%) optical density. Within the salt conditions, we found a stronger growth reduction with higher salt concentrations in the synthetic defined media (SDM). This growth-inhibitive effect was previously shown for cells grown in rich YPD media [23]. As expected, limitation of glucose in the medium led to a decrease of the maximal density as well. The combination of low glucose (0.5% *w/v*) and 200 mM LiCl did not show a cumulative effect of the individual growth reducing effects.

The difference in cell density between the two experiments (Figure 1a,b) has been observed throughout all our replicates. Therefore, we assume that the difference in culturing technique has a major influence. The cultures in Figure 1 b are grown in a 50 mL Falcon tube format and thus most likely are aerated more efficiently and thus reach higher densities than those in Figure 1a, grown in the honeycomb plate of the Bioscreen.

### 3.2. Cellular Response to Salt Stress

#### 3.2.1. Hog1 Phosphorylation upon Salt Stress

The addition of NaCl to the medium causes a hyperosmotic shift, which results in water efflux from the cell, hence volume reduction [24]. The HOG pathway of *S. cerevisiae* governs the adaptive process to changes in microenvironmental osmolarity [25].

During the adjustment to hyperosmotic conditions, Hog1, the central kinase of the pathway, becomes phosphorylated, which leads to activation of Hog1-repressed genes, such as those essential for glycerol accumulation [26]. Thus, the phosphorylation status of Hog1 is a good readout of yeast adjustment to hyperosmotic shock in yeast.

To characterise the HOG response upon LiCl in comparison to NaCl, we measured the temporal phosphorylation pattern of the Hog1 protein upon exposure to different salt concentrations.

**Figure 2:**
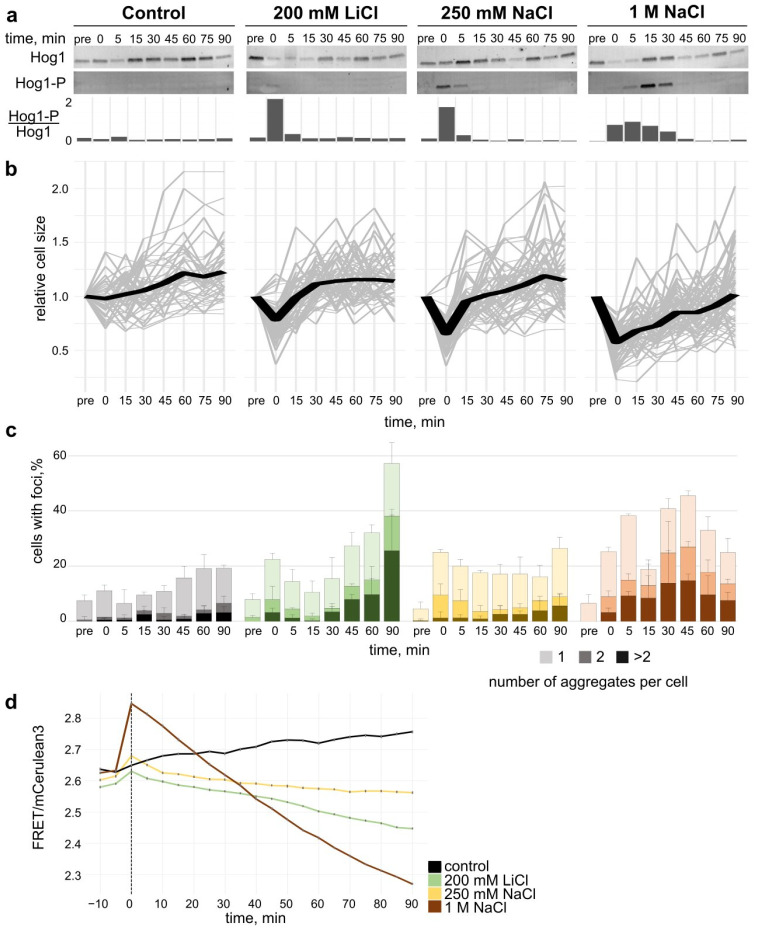
Assessment of impact of salt stress on yeast cells. (**a**): Activation of the HOG pathway upon salt stress. Western blot of Hog1 phosphorylation in *S. cerevisiae* before and up to 90 min after addition of salt stress. Hog1-P/Hog1 is the ratio of the Hog1 phosphorylation signal divided by the signal for the total Hog1. (**b**): Cell volume recovery upon osmotic upshift. Relative cell size was characterised by change in circumference of the cells observed in the microscope. Single cell trajectories (grey) and the average (black) are shown. (**c**): Salt stress induced aggregation in yeast cells. Percentage of cells with Hsp104-GFP foci before and after application of salt stress. Colour shades represent the number of aggregates per cell; at least 100 cells per timepoint and condition were assessed. Error bars indicate SD (**d**): NaCl and LiCl stress recovery and crowding. FRET/mCerulean3 ratiometric plot before and after salt upshift (vertical line). Cells were imaged every 5 min for 10 min prior and 90 min after the media exchange to the standard SDM (grey), 1 M NaCl (red), 250 mM NaCl (yellow), and 200 mM LiCl (green). One out of three representative experiments is shown. Error bars indicate SEM.

As expected, no upshift in phosphorylation was observed in the absence of salt stress (Figure 2a). 1 M NaCl led to a rapid increase in the phosphorylation of Hog1, observed immediately after the salt was applied. The phosphorylation level remained high up to 30 min, followed by a progressive decrease from the 45 min timepoint (Figure 2a bottom and Supplementary Figure S1b). A reduction in phosphorylation signal 90 min after NaCl application was detected, similar to pre-salt stress level. 250 mM NaCl upshift resulted in a higher initial Hog1 phosphorylation status right after application of the stress than in case of 1 M NaCl. However, after 5 min, the phosphorylation status returned to pre-stress and remained unchanged from 15 min. The lower activation level and prolonged response time for the severe hyperosmolar shift by 1 M NaCl in comparison to lower salt amounts has been described before [24].This is consistent with previous studies, which have shown that the strength and timespan of Hog1 phosphorylation is dependent on the strength of the osmotic upshift [18]. Addition of 200 mM LiCl led to an immediate phosphorylation of Hog1, which quickly returned to non-phosphorylated status 5 min after the salt upshift. This pattern is similar to that observed for 250 mM NaCl.

Taken together, the osmotic response via the HOG pathway is comparable in strength and length for 200 mM LiCl and 250 mM NaCl, which is consistent with previous reports that the osmolality of the salt is the dominant factor for the Hog1-dependent hyperosmotic salt stress response [27].

#### 3.2.2. Cell Volume Recovery upon Salt Stress

Upon osmotic upshift, water is drawn from the cell, leading to a loss of cell volume and an increase in macromolecular density (macromolecular crowding or MMC) [28,29].

We determined the cell volume of yeast cells by tracking their boundaries over time. For cells growing in standard conditions without salt stress, we observed a steady increase of the average cell size of about 20% over a time span of 90 min, which illustrates normal vegetative cell growth (Figure 2b). An upshift of 1 M NaCl in the growth medium led to an immediate decrease in cell volume to approximately 50% of the original size. Over 90 min cells recovered to their initial volume in a linear manner. When only upshift of 250 mM NaCl was applied, the cells volume was rapidly reduced by about 40% on average. After 15 min, the cells recovered on average to 95% of their original cell volume. At timepoints of 60 min–90 min, we observed vegetative growth. An addition of 200 mM LiCl led to a reduction of cell volume of 25% of the initial volume on average, which was fully restored 15 min after. From 30 min onwards, the cells exhibited vegetative growth.

#### 3.2.3. Formation of Hsp104 Aggregates upon Hyperosmolar Salt Shift

We used fluorescence microscopy to determine whether addition of LiCl or NaCl leads to aggregation of proteins in yeast cells (Figure 2c and Supplementary Figure S2). Over a timespan of 90 min, we followed the formation of aggregates via the GFP-tagged protein disaggregase Hsp104 (hereafter named Hsp104 aggregates) following salt stress. Hsp104 has been used in multiple studies to indicate the frequency and localisation of aggregates in yeast cells due to its ability to bind misfolded proteins [30,31].

In 200 mM LiCl-exposed cells, the fraction of cells with Hsp104 aggregates was doubled to about 20% upon salt application with a further increase starting after 30 min up to 60% cells with aggregates at 90 min.

An osmotic upshift with 250 mM NaCl led to an immediate formation in about 20% of the observed cells compared to the control conditions and over the time of 90 min, this percentage remained largely unchanged.

1 M NaCl-treated cells also showed an initial increase, which was detected up to 5 min after start of the experiment, before decreasing at 15 min. A subsequent increase to over 40% cells with aggregates followed at 45 min after addition of salt with a repeated drop in fraction of cells with aggregates towards the end of the experiment.

Cells treated with salt exhibited aggregate accumulation in the early timepoints. While for 250 mM NaCl and 200 mM LiCl, which have a similar osmolality, the initial aggregate formation seems comparable, LiCl seems to also cause secondary aggregation on a longer time scale. The exposure to 1 M NaCl showed a stronger and more prolonged aggregate accumulation.

#### 3.2.4. Crowding Modulation upon Salt Stress

To investigate how crowding levels change upon hyperosmotic stress correlatively to observed cell volume changes, we used yeast cells expressing aFRET-based sensor, termed crGE, allowing for acute macromolecular crowding quantification [17] (for details, see Supplementary Figure S3).

We observed a clear increase of crowding immediately upon stress exposure for all conditions tested (Figure 2d). These observations are consistent with the immediate salt-induced cell volume shift. In the control condition where no salt stress was applied, we observe a light increase of ratiometric FRET values measured between 0 to 90 min.

Upon immediate exposure to 1 M NaCl, the FRET ratio increased by approximately 0.2. 250 mM NaCl and 200 mM LiCl showed a smaller increase in FRET efficiency, a shift coherent with the equivalent osmolality effect. This correlates with the strength of the cell size reduction observed with a high concentration of salt as water flows out the cells under the associated strong hypertonic condition (Figure 2b,d).

Ninety minutes after stress exposure, as the cells progressively recovered to their initial volume, macromolecular crowding decreased. Ratiometric FRET for all stress tested reached lower levels than prior to stress application, thus reflecting a lower crowding environment than initially measured. Equally, this progressive crowding reduction was observed to a greater extent after 1 M NaCl exposure than the two other salt conditions. 250 mM NaCl and 200 mM LiCl displayed a similar behaviour in the first 40 min after shock with MMC being reduced over time.

### 3.3. Effect of Salt Pre-Adaptation on Formation of Protein Aggregates

**Figure 3:**
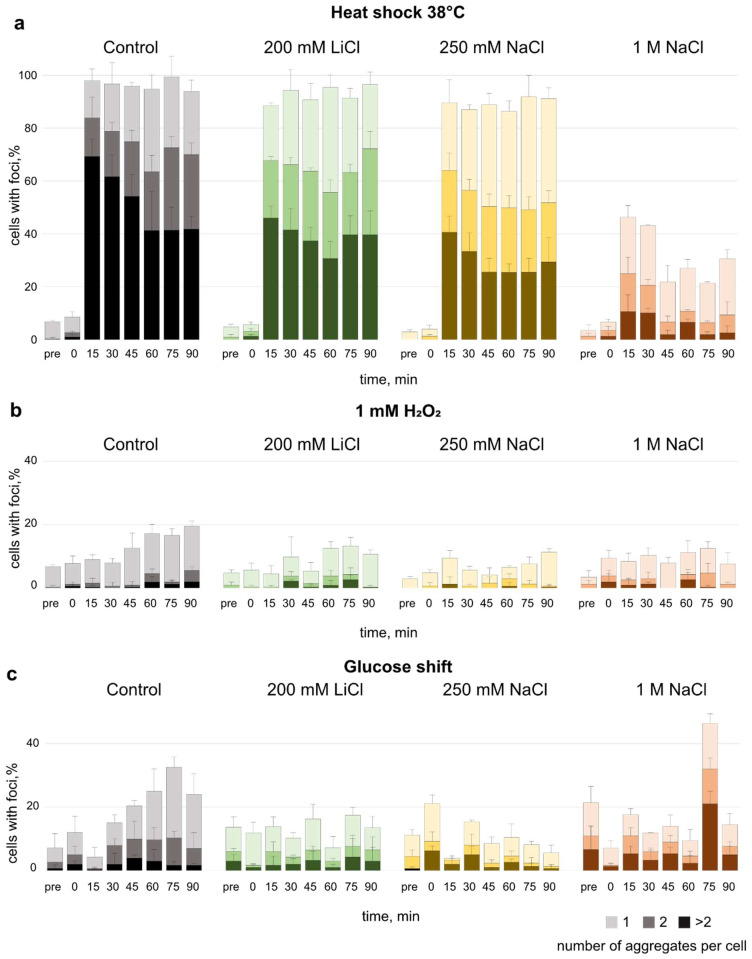
Percentage of cells with Hsp104-GFP foci after two hours of pre-adaptation to salt, followed by the exposure to (**a**) heat shock, (**b**) hydrogen peroxide, or (**c**) shift to low glucose SDM (0.05% glucose (*w/v*)). Cells were fixed in 3.7% (*v/v*) formaldehyde for imaging. Colour shades represent the number of aggregates per cell, error bars indicate SD.

To investigate if the pre-adaptation to different salts has an influence on the stress-induced protein aggregation, we examined the formation of Hsp104 foci (Figure 3 and Supplementary Figure S4). Yeast cells expressing this aggregate marker tagged with GFP were observed during diverse stresses after cultivation in sodium chloride or lithium chloride.

Heat shock 38 °C led to a strong increase in the fraction of cells that displayed Hsp104 aggregates (Figure 3a). Notably, the accumulation of Hsp104 aggregates increased to a fraction of almost 100% for cells in control medium, 200 mM LiCl, and 250 mM NaCl after 15 min of applying the heat shock. Cells grown in 1 M NaCl only showed an increase to about 40% of the cells exhibiting Hsp104 aggregates after 15 min and around 25% during later timepoints.

In cells treated with hydrogen peroxide after pre-adaptation to salt, the extent to which Hsp104 aggregates formed was much lower compared to cells subjected to heat shock (Figure 3b). Overall, it seems that the concentration of the applied salt has a stronger effect on the prevalence of Hsp104 aggregates in heat shocked cells than in cells exposed to hydrogen peroxide.

Caloric restriction is associated with longevity in several phyla [32]. A shift to low glucose in yeast has been shown to promote the long-term survival and increase the number of daughter cells produced by a mother cell [32]. It has been reported that lithium can prolong life span in several organisms, but this has not been shown for *S. cerevisiae* [16]. Therefore, we tested whether pre-adaptation to lithium chloride and a following shift to low glucose affect the formation of aggregates and compared it to NaCl to see if any observed effects would be specific to LiCl (Figure 3c).

Glucose depletion in the control condition showed a moderate upshift in the formation of aggregates. There was no significant effect from preadaptation to LiCl or 250 mM NaCl. However, in cells exposed to 1 M NaCl, there was a strong foci accumulation observed at 75 min of glucose downshift. Overall, the accumulation of aggregates in cells exposed to low glucose appears to fluctuate over the observed time period. In sum, the strongest effect on Hsp104 aggregate accumulation can be seen in cells exposed to heat shock after adapting to 1 M NaCl.

### 3.4. Effect of Lithium Chloride on Long-Term Survival

**Figure 4:**
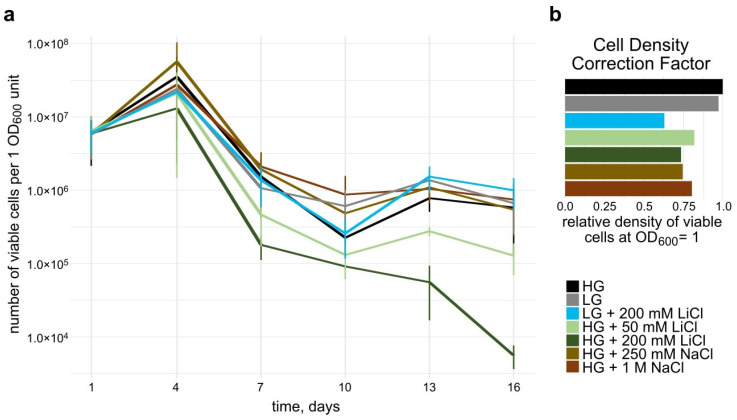
Long-term viability and Cell Density Correction Factor (CDCF) of *S. cerevisiae* strain BY4741 grown in SDM containing different concentrations of salt and glucose (HG = high glucose, 2% (*w/v*) glucose, LG = low glucose, 0.5% (*w/v*) glucose). (**a**): Survival assay. Cells were inoculated into salt medium at day 1 and viability was measured subsequently every three days by plating and counting colony forming units. The values have been corrected by the Cell Density Correction Factor (CDCF) calculated for each specific condition. Error bars represent SD. Log scale on Y axis. (**b**): Cell Density Correction Factor. Relative density of viable cells at OD₆₀₀ = 1 on day 1 of the survival assay compared to the High Glucose (HG) condition. A lower CDCF indicates fewer viable cells at the same OD₆₀₀.

To test long-term viability of the yeast cells under the influence of different concentrations of salt and glucose, we performed a survival assay (Figure 4a,b). Caloric restriction, as well as growth in medium with high osmolarity have been shown to support the long-term survival of budding yeast [33,34,35].

To assess whether the difference in culturing conditions affects the OD₆₀₀ measurements, we correlated cell density and OD₆₀₀ for each condition on day 1 (Figure 4b). At the same OD₆₀₀, cells grown with only 0.5% glucose showed almost the same density of viable cells as the control condition at 2% glucose. Addition of LiCl or NaCl led to a reduction in the number of cells to about 70–80% compared to the control condition.

While in all growth conditions, viable cell count increased by day 4, there was a steep decrease from day 4 to day 7 for all conditions. We observed that long-term viability at day 16 for cells grown in SDM containing 1 M and 250 mM NaCl and SDM supplemented with 0.5% glucose was similar to cells grown in standard SDM. Analysis of relative viability showed the same trends (Supplementary Figure S5).

The addition of 50 mM LiCl to the medium caused a decrease in viability, that furthermore declined in cells exposed to 200 mM LiCl in the medium.

However, growing cells in SDM containing only 0.5% glucose and 200 mM LiCl mitigated the effect observed for 200 mM LiCl in 2% glucose and restored long-term viability to the same level as the conditions without LiCl.

## 4. Discussion

Recently, lithium treatment was shown to extend the lifespan of several organisms [16] and promote recovery following stress [36]. Similarly, a lifespan extending effect by glucose limitation has been shown when cultivating the yeast *S. cerevisiae* at 20 °C in YPD medium [37]. However, the potency and mechanism of LiCl on the yeast cell life span is yet to be determined.

In this work, we show that under normal conditions, high concentration of LiCl is detrimental to long-term survival of cells. This effect can be mitigated by growing cells under glucose limitation. We further revealed that at comparable molality, lithium causes a similar osmotic upshift response as NaCl. Finally, we show that cells are more prone to protein aggregation during live cell imaging in the presence of LiCl compared to NaCl. Further, adaptation to high concentration of NaCl reduces the fraction of cells with aggregates upon heat shock.

Hyperosmolar shock causes the formation of aggregates in yeast, which we visualised by monitoring a chaperone, Hsp104. Salt concentrations of 200 mM LiCl and 250 mM NaCl result in a similar osmolality upshift, yet we observed a strong increase in aggregation frequency after the initial response to hyperosmolar shift of the medium for LiCl (Figure 2b,c). Therefore, we postulate that an effect specific to the Li^+^ ions might mediate this increased aggregate formation. This could be due to effects on the membrane potential. The consequences of this would result in a change in energy homeostasis, dysfunction of transmembrane transporters, and modifications of cell wall stability. However, more experiments are required to confirm this. From this we conclude that protein aggregation caused by LiCl exposure is the result of additional factors beyond osmotic properties.

We postulate further that a decrease of the intracellular macromolecular crowding (MMC) under salt addition might be a long-term adaptation to the presence of higher salt concentrations in the environment, which continues after completion of the initial osmotic upshift response. This change in MMC could indicate an increase in cell size in response to higher ionic concentrations in the environment of the cell, thus a change in the surface to volume ratio of the cell. The effect of Li^+^ ions on the mannan–protein complex of the cell wall have been described previously, and have been linked to the standard lithium acetate (LiAc) transformation protocol used for yeast [38,39]. The combination of changes in cell wall structure and cellular macromolecular density could lead to a difference in optical properties of the cells. Hence, this could explain the difference in cell density per OD₆₀₀ unit of cells grown in media with different salt compositions and could be a factor that should be considered when performing long-term survival studies.

In our study, accumulation of Hsp104 aggregates following heat shock was reduced in cells pre-adapted to 1 M NaCl compared to cells in all other conditions (Figure 3a). High salt concentration could induce an initial pre-adaptation that subsequently enables the cell to an increased resistance if a secondary stressor is applied, meaning that in this case we observe fewer Hsp104 aggregates. It has been previously reported that different types of stress can confer a cross protection towards each other [40,41]. However, our findings could also hint to a salt-dependent mechanism either interfering with effective Hsp104-GFP recruitment and thus the labelling of protein aggregates or faster aggregate clearance. The less-frequent accumulation of Hsp104 aggregates upon heat shock in cells adapted to 1 M NaCl indicates an influence on the protein quality control (PQC). Further investigation could address the involvement of different components of the PQC contributing to the dynamics of aggregates formed during salt stress, as well as their dependency on ATP and provide more information on spatial sorting of aggregated proteins under the influence of salt.

While in heat-shocked cells the concentration of sodium chloride has a distinct effect on Hsp104 aggregation, this is less prominent in cells subjected to hydrogen peroxide. Proteins are affected differently during heat shock (physical) or oxidative stress (chemical), which might cause differences in the amount and type of misfolded proteins, as well as the dynamics of aggregate formation [42]. During oxidative stress, one of the many events that happen in the cell is the depletion of ATP [43], which results in reduced disaggregase activity of Hsp104 [44,45]. We hypothesise that this is the reason why less Hsp104 aggregates can be observed under hydrogen peroxide exposure, and the preadaptation to salt does not seem to play a role either.

Lithium has been shown to associate with ATP-Mg^2+^ and could thus influence how enzymes bind to ATP. While still allowing signalling through purinergic receptors, lithium bound ATP-Mg^2+^ could also potentially be recognised by enzymes and initiate signalling [46]. Enzymes might be unable to utilise ATP-Mg2+ in its LiCl bound form, which could be an explanation for the high accumulation of aggregates in lithium chloride treated cells (Figure 2c), as ATP depletion has been shown to hamper the effective clearance of protein aggregates by Hsp104 [44].

Monovalent cations can form a complex with macromolecules, such as proteins and DNA [46,47,48,49,50]. Specifically for lithium, there are hints that the association with ATP as well as the displacement of cations like Na^+^ from allosteric binding sites of G-protein coupled receptors (GPCRs) [51] or sodium-dependent neurotransmitter transporters [52] could influence neurotransmission and explain some of its efficacy as a drug for the treatment of bipolar disorders [53].

We expected low glucose media, as well as 250 mM and 1 M NaCl would increase lifespan, in comparison to standard conditions. However, viability did not seem to be affected by these conditions in our settings. Rather, we observed that the combination of low glucose medium and 200 mM LiCl had a noticeable effect on the long-term survival of cells, as opposed to cells that were grown in standard conditions supplemented with 200 mM LiCl. Studies on *S. cerevisiae* suggest a metabolic effect of lithium by inhibiting kinase activity of phosphoglucomutase and inositol monophosphatase [9,10]. Inositol has been at the centre of research to understand the pharmacology of lithium in patients that are affected by bipolar disorder or neurodegenerative disease [54]. Both these enzymes are linked to the central carbon metabolism. Furthermore, a link between inositol phosphate synthase, the glucose response pathways, and the fatty acid synthesis in yeast has been suggested [55], as well as a link to normal mitochondrial function and autophagy, both implicated in various human diseases and normal cell physiology [56].

Yeast metabolism differs based on the available energy source and kinase inhibition by LiCl. This might be a key reason why growth under low glucose conditions mitigates the toxicity of LiCl observed in high glucose conditions [9,57]. Our results suggest a possible role of lithium in glucose metabolism that might affect longevity while at the same time leaving the ability of the cell to respond to a second stressor unaffected. These aspects are relevant also in various human diseases, including neurodegenerative, mood, and metabolic disorders.

## Data Availability

Raw data from this study are available on reasonable request from the corresponding author.

## Supplementary Materials

The following are available online at https://www.mdpi.com/article/10.3390/microorganisms10030590/s1, Supplementary Figure S1: Activation of the HOG pathway upon salt stress. Supplementary Figure S2: Microscopy images of aggregate formation upon salt stress. Supplementary Figure S3: A: The crGE mCeruelan3-mCitrine sensing crowding sensor upon salt stress.
