# Supplementary material for: The Effect of Lithium on the Budding Yeast Saccharomyces cerevisiae upon Stress Adaptation
Source: Microorganisms. 2022 Mar 9;10(3):590. doi: 10.3390/microorganisms10030590 (PMC8953283; doi:10.3390/microorganisms10030590)
Supplement: Supplementary file 1 [file microorganisms-10-00590-s001.zip › microorganisms-1537736-supplementary.pdf]

microorganisms

MDPI

Supplementary material

The effect of lithium on the budding yeast Saccharomyces cerevisiae upon stress adaptation

Patrick Reith[1,2,3], Svenja Braam[1,2], Niek Welkenhuysen[1,2], Sarah Lecinski[4], Jack Shepherd[4,5], Chris MacDonald[5], Mark Leake[4,5], Stefan Hohmann[3], Sviatlana Shashkova[1,2,§], Marija Cvijovic[1,2,*]

[1] Department of Mathematical Sciences, University of Gothenburg, Gothenburg, Sweden
[2] Department of Mathematical Sciences, Chalmers University of Technology, Gothenburg, Sweden
[3] Department of Biology and Biological Engineering, Chalmers University of Technology, Gothenburg, Sweden
[4] Department of Physics, University of York, York, YO10 5DD, United Kingdom
[5] Department of Biology, University of York, York, YO10 5DD, United Kingdom
§ Current address: Department of Physics, University of Gothenburg, 412 96 Gothenburg, Sweden
* Corresponding author: marija.cvijovic@chalmers.se

Supplements

Citation: Reith, P.; Braam, S.; Welkenhuysen, N.; Lecisnki, S.; Shepherd, J.; McDonald, C.; Leake, M.C.; Hohmann, S.; Shashkova, S.; Cvijovic, M. The Effect of Lithium on the Budding Yeast *Saccharomyces cerevisiae* upon Stress Adaptation. *Microorganisms* **2022**, *10*, 590. https://doi.org/10.3390/microorganisms10030590.

Academic Editor: Nicoletta Guaragnella, Anita Krisko and Tiago Fleming Outeiro

Received: 17 December 2021
Accepted: 7 March 2022
Published: 9 March 2022

Publisher's Note: MDPI stays neutral with regard to jurisdictional claims in published maps and institutional affiliations.

Copyright: © 2022 by the authors. Licensee MDPI, Basel, Switzerland. This article is an open access article distributed under the terms and conditions of the Creative Commons Attribution (CC BY) license (https://creativecommons.org/licenses/by/4.0/).

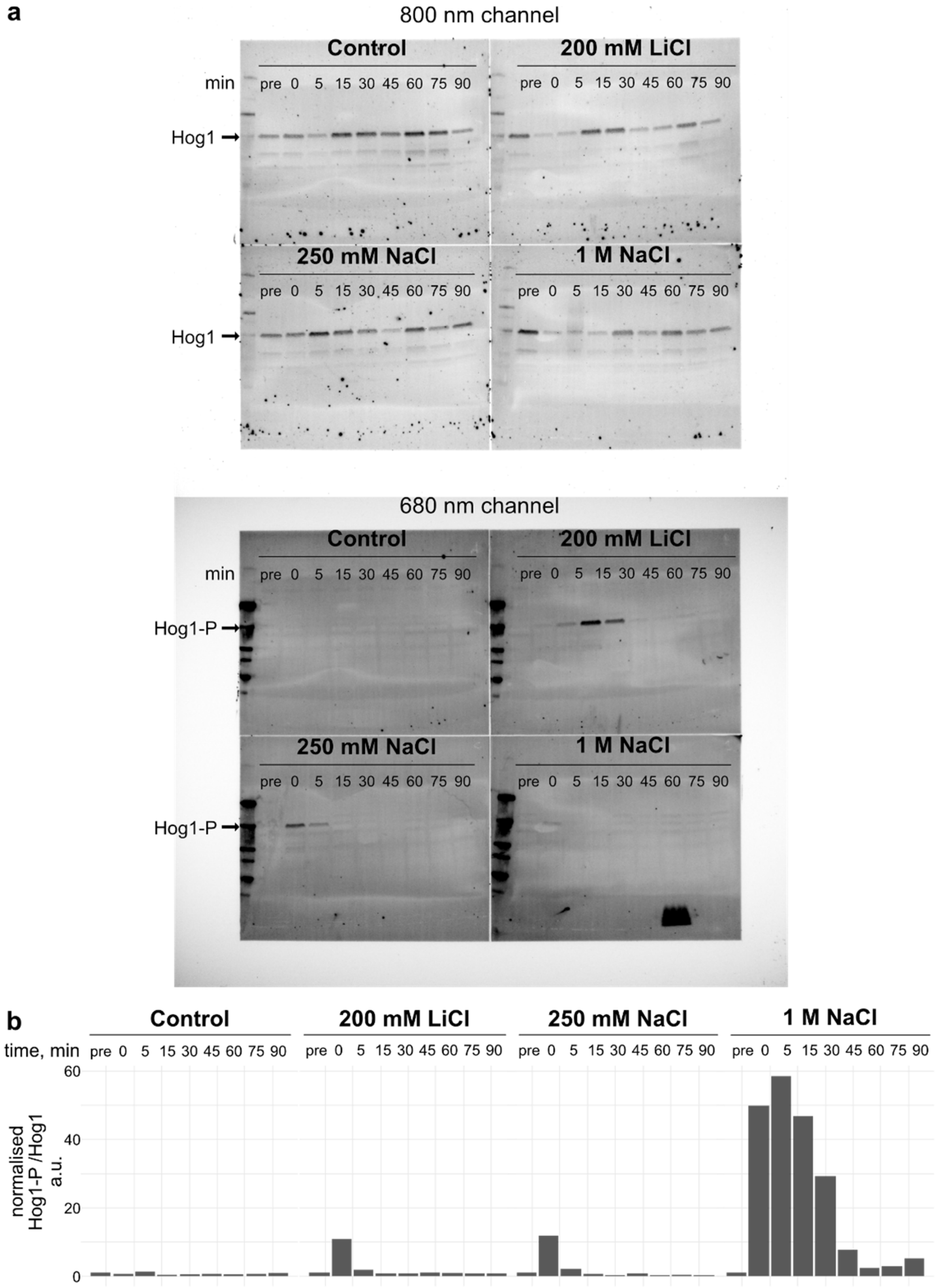

Supplementary Figure S1: Activation of the HOG pathway upon salt stress. Full scale images of Western blot and normalized Hog1 phosphorylation levels. (**a**) Western blot of Hog1 phosphorylation in *S. cerevisiae* before and up to 90 min after addition of salt stress for the 800 nm and the 680 nm channel. (**b**) Normalized Hog1-P/Hog1 is the ratio of the Hog1 phosphorylation signal divided by the signal for the total Hog1 normalized to the pre-stress Hog1-P/Hog1 ratio in arbitrary units.

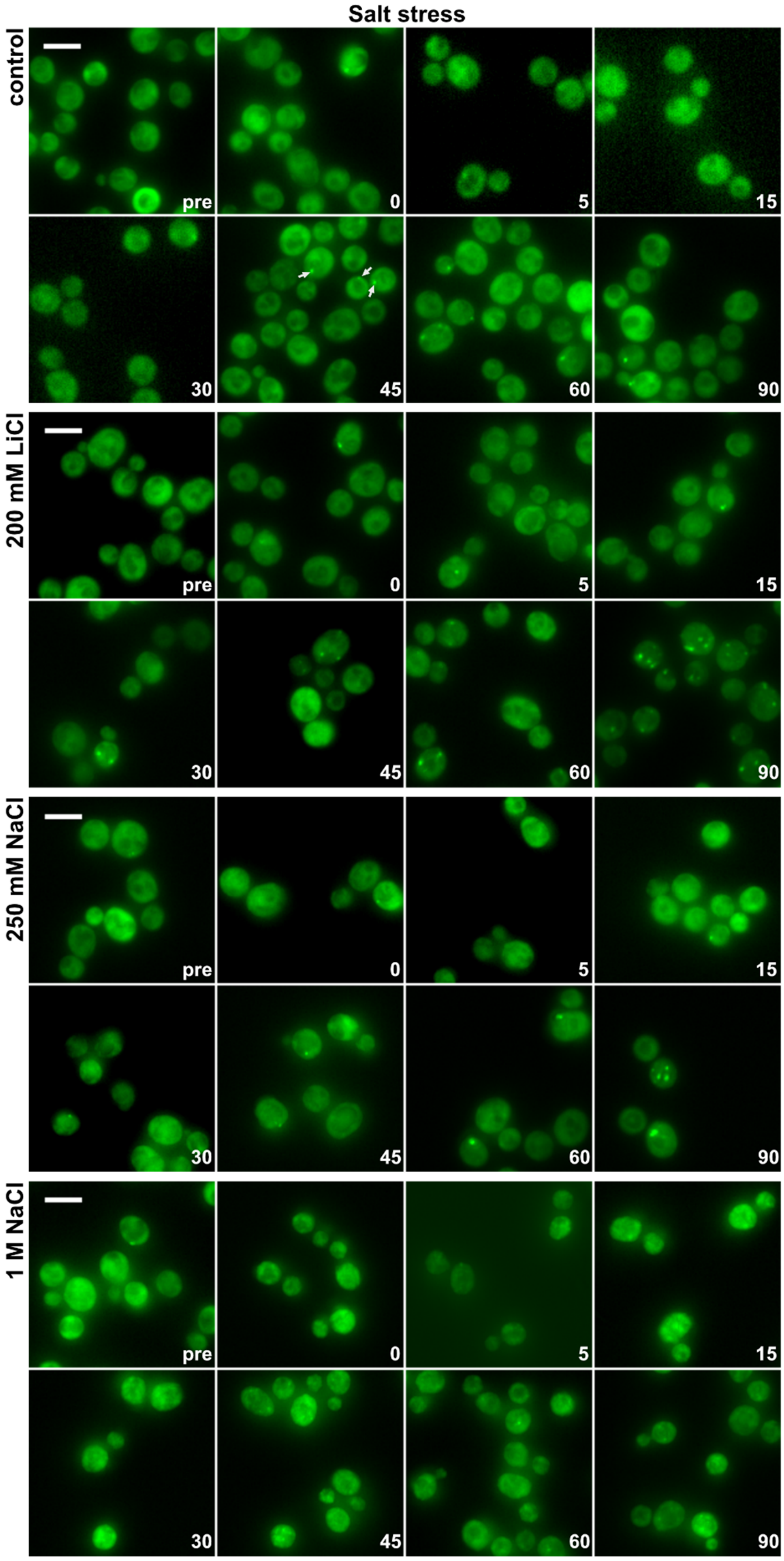

Supplementary Figure S2: Microscopy images of aggregate formation upon salt stress, timepoints in minutes. The contrast was adjusted for representation purposes. Scale bar 5 µm. Arrows in the Control 45 min panel exemplary depict foci counted as aggregates.

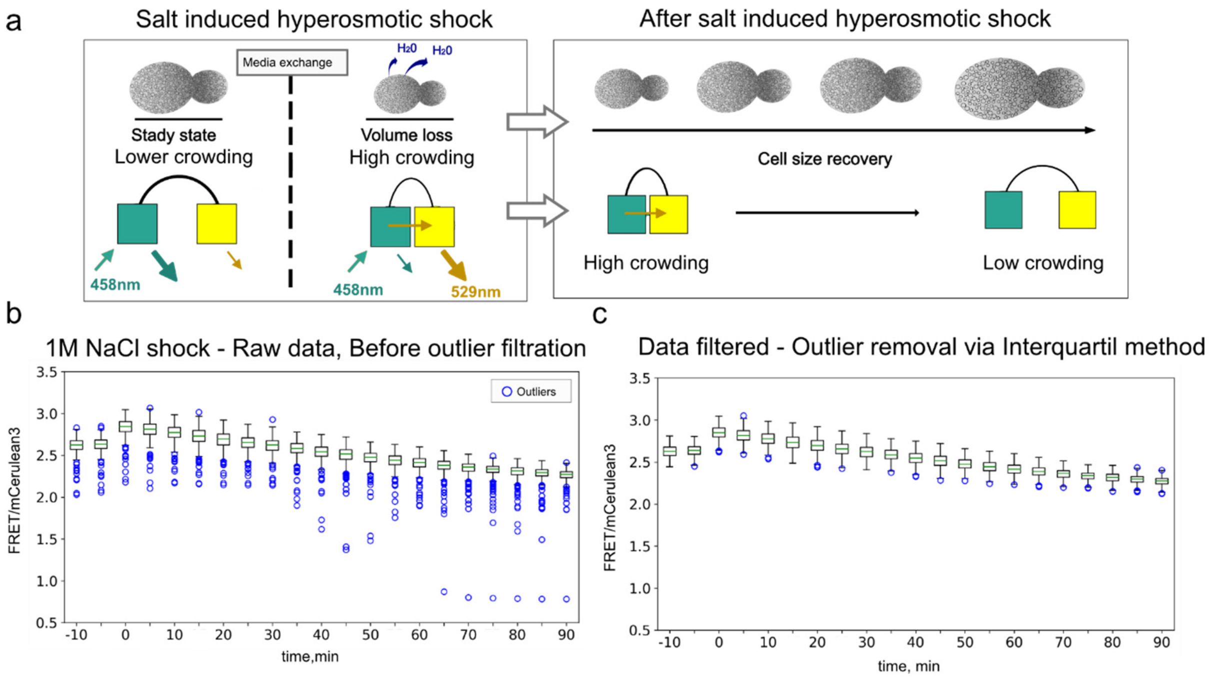

Supplementary Figure S3: (a) The crGE mCeruelan3-mCitrine sensing crowding sensor upon salt stress. Schematic model describing the sensor and the influence of salt induced hyperosmotic shock on cell volume and crowding [61]. The sensor is made by the mCerulean3-mCitrine (donor-acceptor) fluorescent FRET pair, and linked by a flexible domain, the crGE sensor oscillate between a close (high crowding, high FRET efficiency) and open (low crowding, lower FRET efficiency) conformation depending on the intracellular crowding state and giving a distinctive fluorescent readout, the FRET efficiency or ratiometric FRET. **(b-c)** Outliers detection and removal using interquartile methods. Example on cells exposed to 1 M NaCl. **(b)** Boxplot of the same data, output from in-house Python3 utility. Boxes shows the interquartile range (IQR), with bars extending 1.5*IQR from the upper and lower quartile. Blue circles for values considered as outliers. **(c)** Boxplot for filtered data, using in-house Python3 code, values above 1.5*IQR were removed from the analysis.

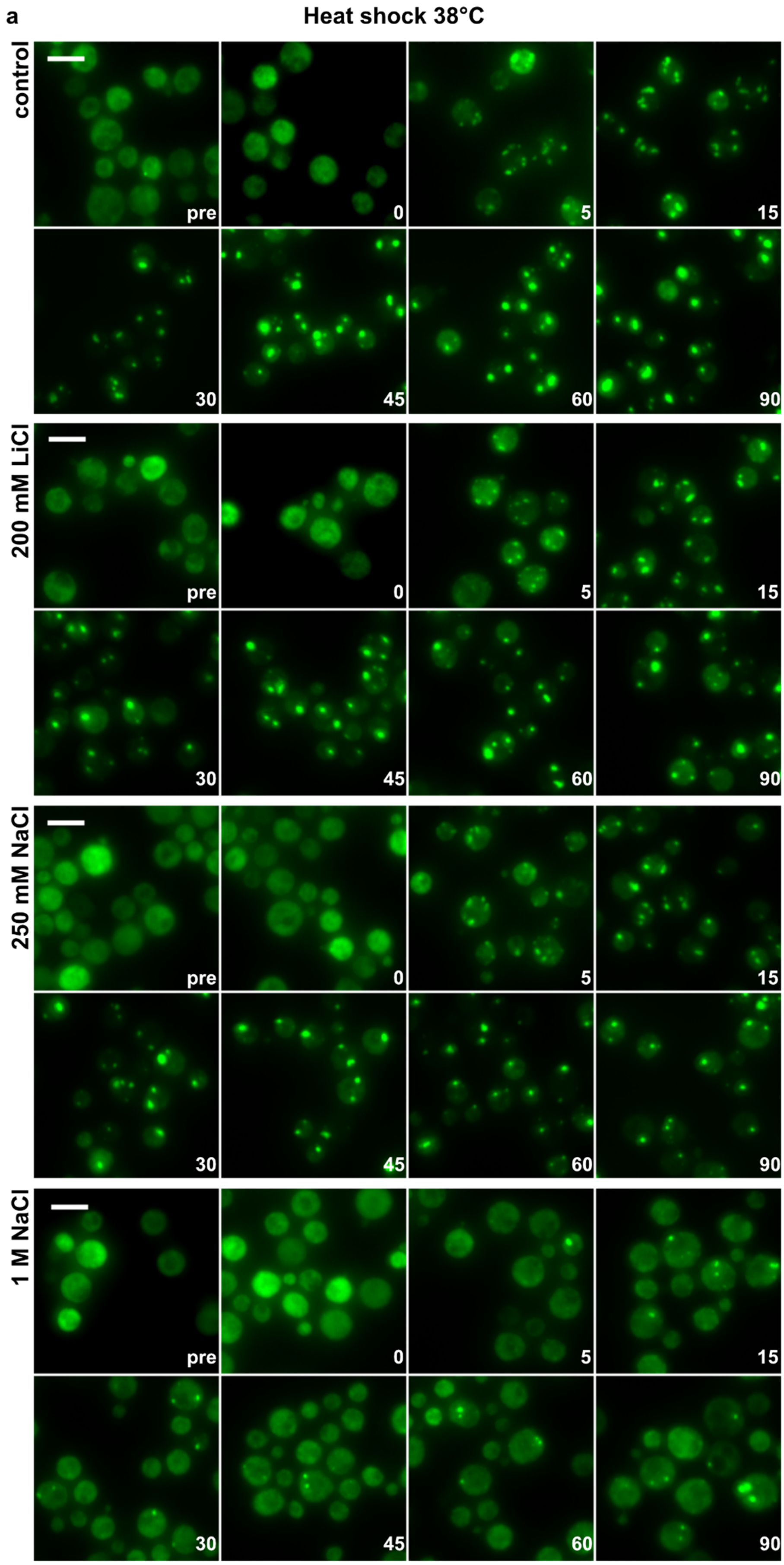
a
Heat shock 38°C
control
200 mM LiCl
250 mM NaCl
1 M NaCl
pre
0
5
15
30
45
60
90

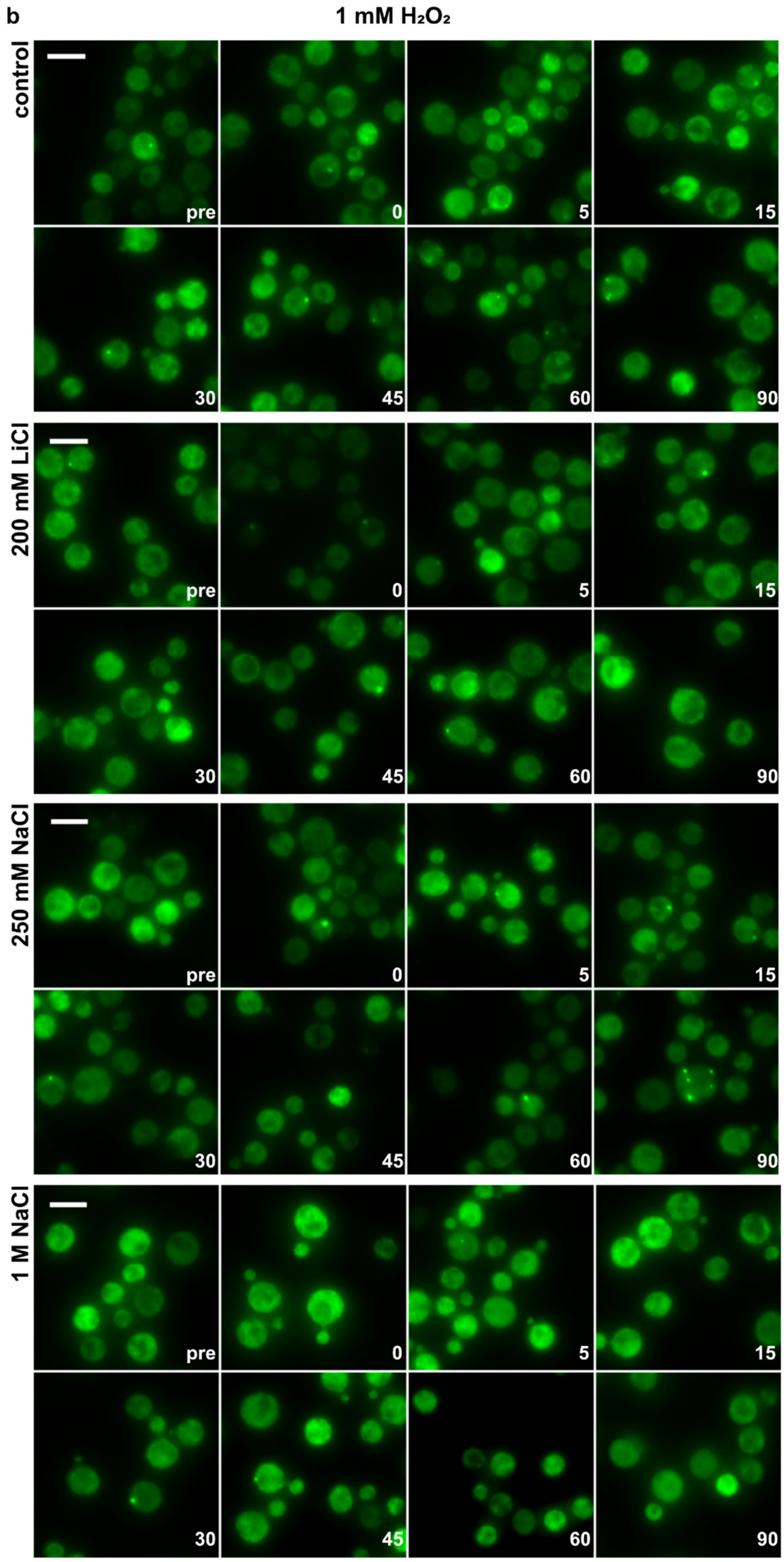
b
1 mM H_2O_2
control
200 mM LiCl
250 mM NaCl
1 M NaCl
pre
0
5
15
30
45
60
90

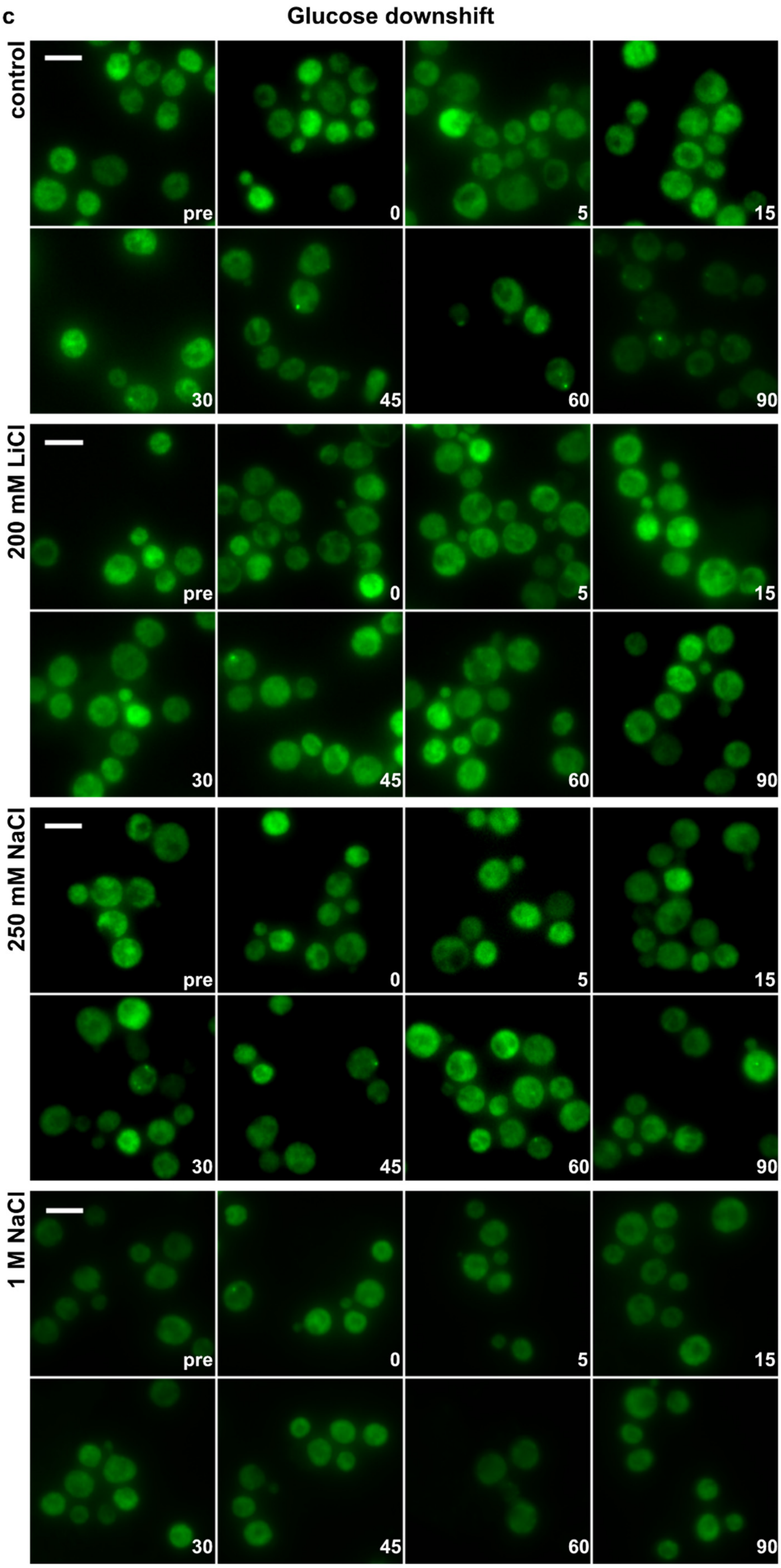
c
Glucose downshift
control
200 mM LiCl
250 mM NaCl
1 M NaCl
pre
0
5
15
30
45
60
90

Supplementary Figure S4: Microscopy images of aggregate formation in salt adapted cells in response to (a) heat shock, (b) H_20_2, (c) glucose depletion, timepoints in minutes. The contrast was adjusted for representation purposes. Scale bar 5 µm.

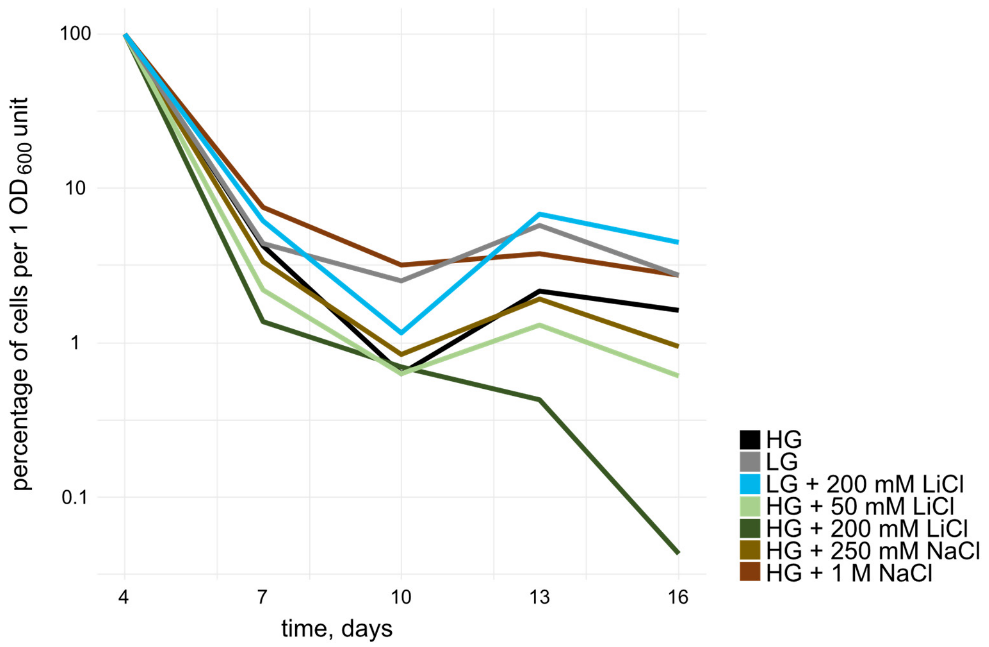

Supplementary Figure S5: Relative long-term viability of S. cerevisiae strain BY4741 grown in SDM containing different concentrations of salt and glucose (HG = high glucose, 2 % (w/v) glucose, LG = low glucose, 0.5 % (w/v) glucose). Survival assay. Cells were inoculated into salt medium at day 1 and viability was measured subsequently every three days by plating and counting colony forming units. The values have been corrected by the Cell Density Correction Factor (CDCF, Figure 4b) calculated for each specific condition. Percentage calculated to averaged maximal cell density observed per condition on day 4. Log scale on Y axis.
